# PreserFlo™ MicroShunt Versus Trabeculectomy in Patients With Moderate to Advanced Open-Angle Glaucoma: 12-Month Follow-Up of a Single-Center Prospective Study

**DOI:** 10.7759/cureus.28288

**Published:** 2022-08-23

**Authors:** Sofia Fili, Kalliopi Kontopoulou, Iraklis Vastardis, Georgios Perdikakis, Markus Kohlhaas

**Affiliations:** 1 Department of Ophthalmology, St. Johannes Hospital, Dortmund, DEU; 2 Department of Ophthalmology, Vista Eye Center, Brugg, CHE

**Keywords:** fibrosis, bleb, anti-glaucoma eye drops, moderate to advanced glaucoma, intraocular pressure

## Abstract

Purpose: The study compares the efficacy and safety of PreserFlo™ MicroShunt (Santen, Osaka, Japan) and trabeculectomy in eyes with moderate to advanced open-angle glaucoma.

Methods: In this prospective study, 300 patients (300 eyes) with moderate to advanced open-angle glaucoma were treated with either PreserFlo MicroShunt or trabeculectomy. The implantation of the PreserFlo MicroShunt was performed in 150 eyes (group A) and trabeculectomy was performed in 150 eyes (group B). The efficacy was evaluated by estimating the absolute and qualified success rates using the Kaplan-Meier survival analysis.

Results: During the 12-month follow-up period, 81.33% of eyes in group A and 94% in group B reduced intraocular pressure (IOP) >20% without glaucoma eye drops. The average IOP 12 months after surgery was 12.89±3.4 mmHg in group A and 11.39±4.5 mmHg in group B, which was significantly lower than the baseline intraocular pressure (group A: 23.47±8.36 mmHg, p=0.00053; group B: 22.03±5.2 mmHg, p= 0.0006). The number of topical medications administered 12 months after ocular surgery was 0.4±0.8 in group A and 0 in group B, compared to 2.5±1.2 in group A and 2.7±0.9 in group B at baseline (group A: p= 0.00091; group B: p= 0.00072). Ten eyes (6.67%) in group A and four eyes (2.67%) in group B were referred for bleb revision due to bleb fibrosis and consequent acute postoperative rise in IOP. Four eyes of group A and two eyes of group B were treated with transscleral cyclophotocoagulation. Five eyes in group B received re-trabeculectomy because of dysfunction of the primary bleb.

Conclusion: PreserFlo MicroShunt reduced the number of antiglaucoma agents compared to baseline, but was inferior to trabeculectomy at reducing IOP after 12 months. Additionally, trabeculectomy produced better absolute success rates after 12 months than PreserFlo MicroShunt in the treatment of moderate to advanced open-angle glaucoma.

## Introduction

One of the leading causes of irreversible blindness worldwide is glaucoma [1,2]. When medical therapy or laser approaches fail to prevent further deterioration of the thickness of optic nerve fibers and degradation of the visual fields, surgical procedures are used to reduce intraocular pressure (IOP). Although the worldwide ophthalmological community still considers trabeculectomy to be the gold standard surgical treatment of glaucoma [3], substantial effort has been made during the last decade to create innovative glaucoma surgical procedures that are as effective as trabeculectomy but with a better safety profile. This has resulted in fewer trabeculectomy procedures in favor of new, microinvasive glaucoma surgery (MIGS) [4,5].

PreserFlo^TM^ Microshunt (Santen, Osaka, Japan) is an innovative, subconjunctival draining minimally-invasive glaucoma device that creates a posterior bleb in the upper subconjunctival space. The PreserFlo Microshunt is composed of poly(styrene-block-isobutylene-block-styrene) (SIBS), a synthetic polymer that was first used in coronary stents, and has shown excellent biocompatibility [6-8], provokes minimal inflammation, and reduces rates of encapsulation compared to other materials used in glaucoma surgery such as silicone rubber and polypropylene [9,10]. The most important innovation of the PreserFlo Microshunt is the self-regulated outflow based on the Hagen-Poiseuille equation, which is achieved because of its 70 μm lumen size, which is considerably smaller than the typical lumen size of tube shunts (300 μm) [7,11].

Although several relatively small studies have shown the efficacy of PreserFlo Microshunt in cases of moderate to advanced primary open-angle glaucoma [5,12,13], we are reporting the results of a head-to-head comparison of PreserFlo MicroShunt and trabeculectomy in a cohort of 300 patients with moderate to advanced primary open-angle glaucoma (POAG). We assessed IOP, local antiglaucoma medication, postoperative complications, and absolute and qualified success rates.

## Materials and methods

This prospective study was conducted in the ophthalmological department of St. Johannes Hospital in Dortmund, Germany. Between September 2019 and September 2021, 300 patients with moderate to advanced open-angle glaucoma were included in this study. A total of 300 eyes underwent either PreserFlo MicroShunt or trabeculectomy. Written consent for surgery was provided by all participants. This study fully respected the tenets of the Declaration of Helsinki [10]. This study was approved by the institutional review board committee of St. Johannes Hospital (13/02/2021/Preserflo).

The participating patients were divided into two groups before surgery: Group A: PreserFlo MicroShunt and Group B: Trabeculectomy. The main inclusion criteria were the following: the diagnosis of moderate or advanced open-angle glaucoma and IOP > 18 mmHg. According to the Hodapp-Parrish-Anderson classification system, moderate to advanced glaucoma patients were defined by visual fields with a mean deviation (MD) worse than -6 dB. The secondary inclusion criteria were the following: cup-to-disc ratio 0.7 to 1.0, IOP higher than the target IOP with maximal tolerated local or systemic medical therapy (two to four antiglaucoma agents or acetazolamide per os). No exclusion criteria are reported for this study. All 300 patients fulfilled the above-mentioned criteria. 

Baseline examinations included best corrected visual acuity (BCVA), slit-lamp biomicroscopy of the anterior and posterior segment, gonioscopy with angle grading, IOP measurement using Goldmann applanation tonometry (GAT), and Humphrey visual field perimetry using Swedish Interactive Testing Algorithm (SITA) Fast. Upon hospital admission, all patients had daily follow-up examinations. The postoperative follow-up visits were conducted at two weeks, one month, three months, six months, and 12 months after the surgical intervention. All patients completed the 12-month follow-up.

Target IOP was set according to the Canadian target IOP Workshop [14]. If this target IOP was not achieved at any time postoperatively, then we restarted local antiglaucoma medication or revised the filtering bleb. In the case of persistently high intraocular IOP that was resistant to the above interventions, we performed additional glaucoma procedures.

Our criteria of absolute success were between 6 mmHg and 15 mmHg, at least a 20% IOP-reduction from the baseline IOP, no use of antiglaucoma agents, and no subsequent glaucoma procedures. As qualified success was defined as an IOP between 6 mmHg and 18 mmHg, at least 20% IOP-reduction from baseline-IOP, the use of fewer antiglaucoma agents than before surgery, and no subsequent glaucoma procedures. An additional glaucoma surgery during the follow-up period was a failure.

One experienced glaucoma surgeon performed the above-mentioned ophthalmological surgeries under general anesthesia on all patients. 

The surgical procedure for the PreserFlo MicroShunt is minimally invasive. A fornix-based subconjunctival and sub-Tenon flap was dissected at the upper nasal/temporal quadrant over a circumference of one to two hours to at least 8 mm posterior to the limbus. After the placement of three mitomycin C-soaked sponges (0.04 mg/ml) under the subconjunctival flap for three minutes, a 3-mm marker marked a point 3 mm away from the border of the surgical limbus. At the distally marked point on the sclera, a 1.2 mm width diamond blade created a small scleral pocket so that the fins of the MicroShunt can seat. A bented 25 gauge needle created a transscleral tunnel from the apex of the scleral pocket into the anterior chamber. The MicroShunt was threaded with bevel up and fins flat into the transscleral tunnel. The fins were then wedged into the scleral pocket. The flow was confirmed visually by wiping the humor drop away and visualizing a small drop. Then an Ologen^TM^ (ProSys International Ltd, London, United Kingdom) implant was placed under the conjunctiva on the scleral flap and the complex of conjunctiva and Tenon capsule were fixated to the limbus with continuous suture (nylon 10-0).

During the trabeculectomy, a fornix-based subconjunctival and sub-Tenon flap was dissected at the upper nasal/temporal quadrant over a circumference of two hours. Then a square lamellar scleral flap of 3 x 3 mm was created with a crescent knife. Two sponges of approximately 1 x 1 cm soaked with mitomycin C 0.04% (0.04 mg/ml) were placed subconjunctivally for three minutes and then thoroughly rinsed out with a balanced salt solution. A paracentesis was placed and a trabeculectomy of about 1 x 1.5 mm was created with the paracentesis knife. Peripheral iridectomy was performed by rinsing the pigment sheet and then fixing the loose scleral flap with two single sutures (10-0 nylon). Then an Ologen implant was placed under the conjunctiva on the scleral flap and the conjunctiva and the Tenon capsule were fixated to the limbus with continuous suture (nylon 10-0).

Local postoperative therapy in both groups was prednisolone acetate 1% six times daily, moxifloxacin three times daily, and cyclopentolate twice daily, which were gradually tapered. Antiglaucoma agents were not administered postoperatively.

Statistical calculations were performed with IBM SPSS Statistics for Windows, Version 22.0 (Released 2013; IBM Corp., Armonk, New York, United States). We were able to examine whether the selection of PreserFlo MicroShunt or trabeculectomy influenced the efficacy of the intervention with an independent sample t-test. Paired sample t-test was used when each observation in each group was paired with a related observation in the other group. The statistical significance was set at 5% (p=0.05). Additionally, a Kaplan-Meier survival analysis was performed in order to analyze the absolute and qualified success rates.

We also performed also an a priori power analysis using G*Power 3 [15] in order to test the difference between the two independent group means using a two-tailed test with a small effect size (d=0.2), and an alpha of 0.05. A total sample of 300 participants was needed in order to achieve a power of 0.95. Finally, 300 eyes were included in our sample with two equal-sized groups of 150 eyes.

## Results

A total of 110 men and 190 women with a mean age of 71.65±10.2 years were included in our study. We are presenting here 12-month follow-up data. A total of 125 eyes of group A and 131 eyes of group B had moderate or advanced POAG, four eyes of group A had advanced pigmentary glaucoma, 21 eyes of group A and 19 eyes of group B had pseudoexfoliative glaucoma. Meanwhile, 90% of the participants in group A and 70.7% of the participants in group B were pseudophakic, and 36.7% (55 eyes) and 20% (30 eyes) of the participants in groups A and B, respectively, had gone through previous glaucoma operations (Table 1).

**Table 1:** Demographic data, diagnoses, and previous surgeries of patients included in the study. BCVA: best-corrected visual acuity; CD: cup-disc ratio; IOP: intraocular pressure; MD: mean deviation; MMC: mitomycin-C; TS-CPC: transscleral cyclophotocoagulation; Phaco: phacoemulsification; PCL: posterior capsular lens; RNFL: retinal nerve fiber layer; SD: standard deviation; TE: trabeculectomy

Demographic data	Group A: PreserFlo	Group B: Trabeculectomy	P-Value
Female patients	98	92	
Male patients	52	58	
Total patients	150	150	
Range of age (years)	38-91	48-87	
Average age (years)	73.31	68.99	p=0.002
SD age	11.19	9.24	
Right eye	73	79	
Left eye	77	71	
Total eyes	150	150	
Mean baseline MD (dB) ± SD	-11.8±9.28	-12.64±8.32	p=0.39
Baseline IOP (mmHg) ± SD	23.47±8.37	22.03±5.2	p=0.06
Baseline antiglaucoma agents ± SD	2.6±1.17	2.7±0.7	p=0.1
Baseline BCVA (Snellen) ± SD	0.6±0.25	0.6±0.07	p=0.2
Baseline CD ± SD	0.91±0.14	0.89±0.14	p=0.49
Baseline RNFL thickness ± SD	66.06±14.44	65.43±15.72	p=0.053
Operation			
PreserFlo+Ologen+MMC	140	106	TE+MMC+Ologen
PreserFlo+Phaco+Ologen+MMC	8	44	TE+MMC+Ologen+Phaco/PCL
PreserFlo+MMC+ Ologen+Avastin Inj.	1		
PreserFlo+MMC+Ologen+Iridectomy	1		
Total	150	150	
Diagnosis			
POAG	125	131	
Pigmentdispersion glaucoma	4	0	
PXG	21	19	
Total	150	150	
Previous surgeries			
Phaco/PCL	135	106	
Canaloplasty	35	28	
TS-CPC	11	0	
Trabeculectomy	3	2	
Iridectomy	3	0	
Cypass	2	0	
iStent	1	0	

**Figure 1:**
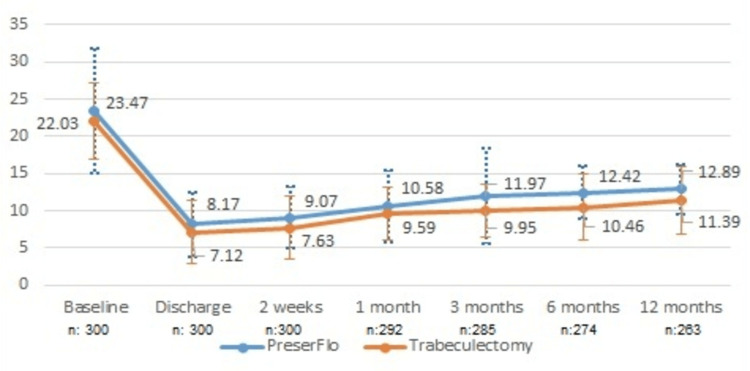
Mean IOP values with standard deviations for the 12-month follow-up period. IOP: intraocular pressure

The mean preoperative IOP was 23.5±8.4 mmHg in group A and 22.03±5.2 mmHg in group B (Figure 1). The mean IOP after one month was 10.6±4.9 mmHg for group A and 9.6±3.5 mmHg for group B and at the sixth postoperative month IOP was 12.4± 3.6 mmHg for group A and 10.5±4.4 mmHg for group B. These IOP-values were significantly reduced compared to those preoperatively (group A: p=0.0009, group B: p=0.00082). At 12 months postoperatively, the IOP remained significantly reduced compared to the preoperative IOP in groups A and B (group A: 12.9±3.4 mmHg, p=0.00053; group B: 11.4±4.5, p=0.0006). The IOP also showed a significant difference between the two groups at the end of the 12-month-follow-up (group A: 12.9±3.4 mmHg, group B: 11.4±4.5, p=0.00151).

**Figure 2:**
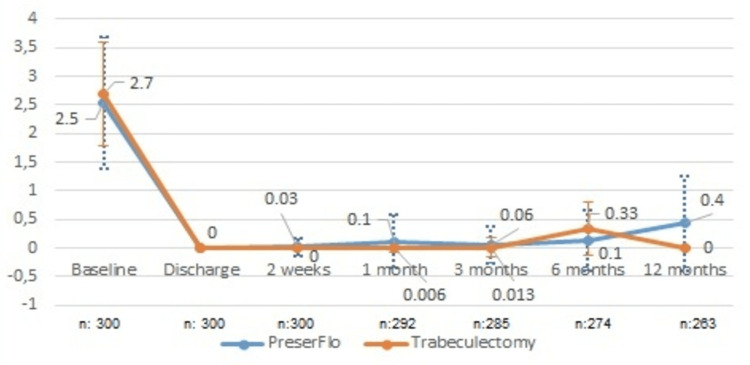
Mean number of local glaucoma agents and the standard deviation for the 12-month follow-up period.

We administered at the sixth postoperative follow-up month an average number of topical medications of 0.1±0.5 in group A and 0.3±0.5 in group B, compared to 2.5±1.2 in group A and 2.7±0.9 in group B preoperatively (Figure 2). At the 12-month-follow-up, topical medication usage remained significantly lower than preoperative usage at 0.4±0.8 medications in group A and 0 in group B. This shows a significantly reduced number of topical glaucoma agents (84%, p=0.00091 in group A; 100%, p=0.00072 in group B).

BCVA was stable between three months and 12 months postoperatively with an average visual acuity of 0.7 in group A and 0.6 in group B. Postoperative BCVA did not differ significantly from preoperative BCVA in either group (group A: p=0.14, group B: p=0.45). No patient in either group had a deterioration in BCVA during the study period.

Both the implantation of PreserFlo MicroShunt and trabeculectomy stabilized all parameters used for glaucoma diagnosis and monitoring including visual fields and optical coherence tomography (OCT) of the optic nerve head. No significant changes were observed in either group in the 12-month follow-up period (Table 2).

**Table 2:** Parameters of monitoring glaucoma progression after PreserFlo™ implantation in group A and trabeculectomy in group B. RNFL: retinal nerve fiber layer; SD: standard deviation

Parameters of glaucoma progression monitoring			
	Baseline	12 months	p-value
Group A			
Visual fields (Mean Value ± SD)	-11.8±9.27	-10.51±8.63	0.17
RNFL thickness (Mean Value ± SD)	66.06±14.44	65.6±12.93	0.4
Group B			
Visual fields (Mean Value ± SD)	-12.64±8.32	-11.4±9.27	0.21
RNFL thickness (Mean Value ± SD)	65.43±15.72	64.52±12.82	0.43

Early postoperative complications (first postoperative month) included a transient ocular hypotony with choroidal detachment in 18 eyes in group A and 22 eyes in group B (Table 3). Ocular hypotony persisted until the sixth postoperative month in one eye of group B. One eye of group B developed a partially compromised function of the filtering bleb with a consequent rise of IOP during the first postoperative month. The increased IOP of this eye was regulated with glaucoma eye drops. A total of 19 eyes of group A and 11 eyes of group B had IOP decompensation because of subconjunctival fibrosis that completely compromised the function of the filtering bleb.

**Table 3:** Postoperative complications after PreserFlo™ implantation (Group A) and trabeculectomy (Group B).

Complications					
	2 weeks	4 weeks	12 weeks	24 weeks	48 weeks
Group A: PreserFlo					
Bulbus hypotony	18	6	0	0	0
Seidel +	1	0	0	0	0
Choroidal detachment	18	5	0	0	0
IOP decompensation	5	6	6	2	0
Group B: Trabeculectomy					
Bulbus hypotony	22	10	1	1	0
Seidel +	0	2	1	0	0
Choroidal detachment	17	8	1	1	0
IOP decompensation	2	0	5	0	4

An acute rise in IOP after the initial glaucoma procedure prompted referral for additional treatment in 18 eyes (12%) in group A and 12 eyes (8%) in group B. Consequently, four eyes in group A underwent needling of the bleb, 10 eyes had a bleb revision, and four eyes had MicroPulse® transscleral cyclophotocoagulation (mTS-CPC) (IRIDEX Corp., Mountain View, California, United States). Compromised function of the bleb resulted in bleb revision in four eyes, re-trabeculectomy in five eyes, transscleral cyclophotocoagulation in two cases, and the implantation of Ahmed valve in one case in group B. Excessive ocular hypotony with choroidal detachment in the first postoperative weeks was treated with the reformation of the AC in two eyes (repetitive AC reformation in one case till the third postoperative month) of group A. The persistent ocular hypotony and coexisting kissing choroidal detachment prompted us to apply compression sutures to the bleb of one of the above-mentioned cases of group A at the end of the first postoperative month. Compression sutures were unsuccessful in this case, and the PreserFlo Microshunt was explanted at the third postoperative month. Excessive postoperative ocular hypotony with persistent choroidal detachment resulted in an AC reformation in eight eyes in group B in the first postoperative month. Three of these cases showed a further persistence of the ocular hypotony at six months, which caused us to apply compression sutures to the bleb (Table 4).

**Table 4:** Additional eye surgeries after PreserFlo™ implantation (Group A) and trabeculectomy (Group B). AC: anterior chamber; mTS-CPC: MicroPulse® transscleral cyclophotocoagulation; TS-CPC: transscleral cyclophotocoagulation

Postoperative Surgical Procedures to Treat Complications					
	2 weeks	4 weeks	12 weeks	24 weeks	48 weeks
Group A: PreserFlo					
Suture removal	1	17	9	4	2
Reformation of AC	2	1	1	0	0
Compression sutures	0	1	0	0	0
AC washout	1	1	1	0	0
Bleb revision	1	5	3	1	0
Needling	2	2	0	0	0
mTS-CPC	1	1	1	1	0
PreserFlo explantation	0	0	1	0	0
Group B: Trabeculectomy					
Suture removal	0	5	5	1	0
Reformation of AC	5	3	0	0	0
Compression sutures	0	0	0	3	0
AC washout	0	0	0	0	0
Needling	0	0	0	0	0
Bleb revision	0	0	2	0	2
Re-trabeculectomy	0	2	1	1	1
mTS-CPC	0	0	0	0	2
Ahmed valve implantation	0	0	0	1	0

**Figure 3:**
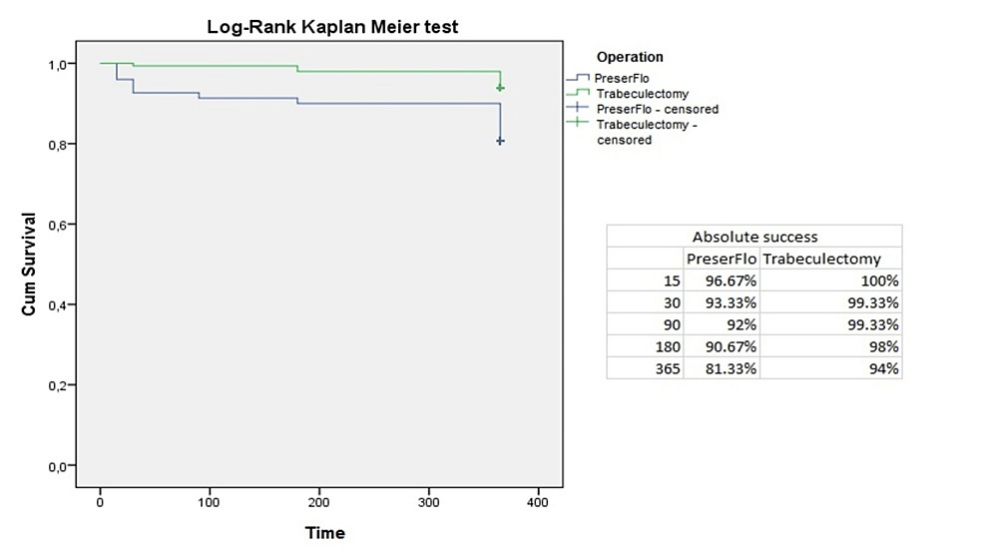
Absolute success criteria in group A (PreserFlo™) and group B (trabeculectomy) presented in Kaplan-Meier survival curves.

**Figure 4:**
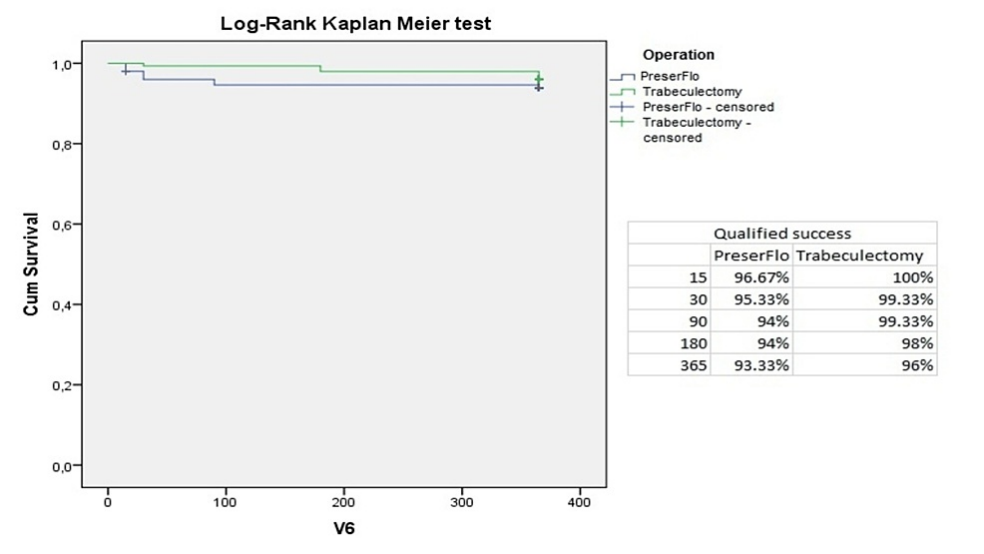
Qualified success criteria in in group A (PreserFlo™) and group B (trabeculectomy) presented in Kaplan-Meier survival curve.

The cumulative probability of absolute success was 96.7%, 93.3%, 92%, 90.67%, and 81.3% in group A and 100%, 99.3%, 99.3%, 98%, and 94% in group B at two weeks and one, three, six, and 12 postoperative months, respectively; p=0.042 (Figure 3). Moreover, we observed a cumulative probability of qualified success of 96.7%, 95.3%, 94%, 94%, 93.3% in group A and 100%, 99.3%, 99.3%, 98%, and 96% in group B at two weeks and one, three, six, and 12 postoperative months, respectively (Figure 4). No statistical difference was identified concerning the qualified success rate between the two groups (p= 0.082).

## Discussion

In our study, the IOP-lowering effect of the PreserFlo MicroShunt is shown to be comparable to that of trabeculectomy, the traditional method of filtration surgery. In both groups, the absolute and qualified success rates were high and remained stable for the entire follow-up period of 12 months. An IOP reduction of at least 20%, a final IOP under 18 mmHg, and fewer topical medications than before surgery was achieved in 93.3% of participants after the implantation of the PreserFlo MicroShunt and in 96% after the trabeculectomy and this reveals no significant difference between the two groups (p=0.09). Contrary to this result, Baker et al. reported lower success rates than ours after both procedures (PreserFlo MicroShunt, 53.9%; trabeculectomy, 72.7%), which differed significantly (p<0.01) from each other [16].

Both the implantation of the PreserFlo MicroShunt and trabeculectomy significantly reduced IOP after six and 12 postoperative months in our cohort, though trabeculectomy reduced IOP significantly more than device implantation. Baker et al. also reported a greater reduction of the IOP in the first postoperative year in the trabeculectomy group (29.1% in the PreserFlo group versus 45.4% in the trabeculectomy group, p<0.01) [16]. Wagner et al. also reported higher IOP with PreserFlo at the end of the sixth postoperative month than in the trabeculectomy group (trabeculectomy, 12.1±7.9 mmHg; PreserFlo, 7.3±7.9 mmHg; p=0.01) [17]. However, experiments in rabbit eyes showed similar postoperative IOP after trabeculectomy and PreserFlo in postoperative weeks four and 12 and implied that the MicroShunt insertion is as effective as trabeculectomy in lowering IOP [18]. Pilunat et al. verified the results of the above experimental study as they showed no significant difference in the reduction of the IOP in the trabeculectomy group compared to the PreserFlo MicroShunt group after six months (average reduction of IOP after six postoperative months in PreserFlo group: 32.1%, in trabeculectomy group: 39.8%, p=0.458) [19].

The results of our study are similar to published results concerning the number of topical glaucoma agents. We showed a significant reduction of the local glaucoma medications of 96% (p=0.00047) and 88% (p=0.00087) in the sixth postoperative month and 84% (p=0.00091) and 100% (p=0.00072) in postoperative month 12 in the PreserFlo group and trabeculectomy group, respectively. Of the patients of the PreserFlo group, 90% were medication-free at the end of the postoperative month 12 and 100% of the patients of the trabeculectomy group were medication-free at the end of the postoperative month 12, and there was no statistical difference between groups (p>0.05). These results are very similar to those of Pillunat et al. who showed that 96.2% of patients treated with PreserFlo and 100% of patients treated with trabeculectomy were medication-free at the end of a six-month follow-up period [19]. Wagner et al. also showed a significant but less substantial reduction in glaucoma medication use at six months with 80% and 83.33% of patients being medication-free in the PreserFlo and trabeculectomy groups, respectively [17]. More patients of both groups required glaucoma medication after 12 months in the Baker et al. study than in others, including ours. They report medication-free rates of 84.8% and 71.6% in the trabeculectomy and PreserFlo groups, respectively [16]. This suggests that the PreserFlo Microshunt reduces glaucoma medication use as effectively as the gold standard glaucoma surgery, trabeculectomy.

A transient ocular hypotony with choroidal detachment occurred in 12% (18 eyes) of the PreserFlo group and 14.7% (22 eyes) of the trabeculectomy group in our study and resolved spontaneously in the first postoperative month. Pillunat et al. reported rates of 15% and 20% in the PreserFlo group and in the trabeculectomy group, respectively [19]. Prolonged hypotony, which required anterior chamber stabilization, was observed in 0.67% (one eye) of the trabeculectomy group in our study, which is lower than 8% (two eyes) found in Pillunat’s trabeculectomy group and 6.1% (eight eyes) of Baker’s trabeculectomy group [16,19]. However, 4.6% of the eyes in Baker’s PreserFlo group also suffered from prolonged ocular hypotony with subsequent choroidal detachment. We found no cases of prolonged ocular hypotony after PreserFlo Microshunt implantation in our cohort over the 12-month follow-up. Patients in our study suffered no serious complications from treatment, which was consistent with other published case series, all of which reported no serious treatment-related complications [16,17,19].

Fibrosis of the filtering bleb with a consequent increase in IOP led to bleb revision or needling in 14 cases (9.3%) and to additional antiglaucoma intervention (mTS-CPC) in four eyes in the PreserFlo group (2.67%). Although a bleb revision was also necessary for 2.67% and additional glaucoma surgery in 5.33% of the trabeculectomy group, no significant difference was detected between the two groups (p>0.05). Baker et al. showed a higher incidence of reoperations after the primary intervention at the end of one year than that in our study (33.9% of the eyes in the PreserFlo group and 19.7% of the eyes in the trabeculectomy group) [16]. However, Pillunat et al. described a higher incidence of new interventions (bleb needling and bleb revision) in the trabeculectomy group (27%) than in the PreserFlo group (4%), which doesn’t agree with previous findings. Nevertheless, our results seem to be superior to the results of other studies and this could be attributed to the stimulation of the subconjunctival fibrosis due to the placement of the Ologen implant under the conjunctiva and Tenon capsule.

Our study demonstrates that IOP can be lowered effectively by both PreserFlo Microshunt implantation and trabeculectomy compared to baseline values. Nevertheless, trabeculectomy reduced the postoperative number of glaucoma medications more substantially than the implantation of PreserFlo Microshunt. Similar results were reported in a study comparing the efficacy of XEN® Gel Stent (Allergan, Irvine, California, United States) with that of trabeculectomy [20]. In both treatment groups, the mean number of IOP-lowering medications was lower; however, after 12 months of follow-up, IOP values were even lower in the trabeculectomy group than in the XEN group. Theiling et al. reported higher absolute (trabeculectomy, 39%; XEN, 33%) and qualified (trabeculectomy, 74%; XEN: 67%) success rates in the 12-month visit [20]. Similarly, the absolute success rate of the trabeculectomy group in our study is higher than those in the PreserFlo group at the end of one year (PreserFlo group: 81.3%, trabeculectomy group: 94%). The implantation of the PreserFlo Microshunt still has superior efficacy (absolute success: 81.3%, qualified success: 93.3% in our study) than that of XEN implantation (complete success: 57.7%; qualified success: 71.1%) at the end of a 12-month-follow-up as the bleb remains longer functional after the wide preparation of the fornix-based subconjunctival and sub-Tenon flap [21].

The main limitation of our study is that we include only a medium-term follow-up duration. Further long-term studies are needed for the evaluation of the long-term efficacy and safety of PreserFlo Microshunt versus trabeculectomy. Our results cannot be generalized to community-based glaucoma practices as the patients in this study had moderate to advanced open-angle glaucoma, which the community-based glaucoma practices would usually refer for specialist treatment.

## Conclusions

In our patient cohort, PreserFlo Microshunt and trabeculectomy are effective in lowering IOP and reducing the local antiglaucoma medication. PreserFlo Microshunt is similarly effective as trabeculectomy in reducing the number of required postoperative antiglaucoma agents. Nevertheless, PreserFlo MicroShunt does not reduce IOP as effectively as trabeculectomy at 12 months. Moreover, trabeculectomy provided better absolute success rates than PreserFlo MicroShunt implantation after 12 months. Postsurgical complications requiring additional interventions occurred in both groups at similar rates.
